# Identification of *Yersinia enterocolitica* isolates from humans, pigs and wild boars by MALDI TOF MS

**DOI:** 10.1186/s12866-018-1228-2

**Published:** 2018-08-17

**Authors:** Katarzyna Morka, Jarosław Bystroń, Jacek Bania, Agnieszka Korzeniowska-Kowal, Kamila Korzekwa, Katarzyna Guz-Regner, Gabriela Bugla-Płoskońska

**Affiliations:** 1 Department of Microbiology, Faculty of Biological Sciences, Institute of Genetics and Microbiology, University of Wroclaw, S. Przybyszewskiego 63/77, 51-148 Wrocław, Poland; 2 Faculty of Veterinary Medicine, Department of Food Hygiene and Consumer Health Protection, Wroclaw University of Environmental and Life Sciences, Norwida 31, 50-375 Wrocław, Poland; 3 Polish Collection of Microorganisms, Ludwik Hirszfeld Institute of Immunology and Experimental Therapy, Polish Academy of Sciences, R. Weigla 12, 53-114 Wrocław, Poland

**Keywords:** *Yersinia enterocolitica*, Pigs, Boars, Zoonotic strains, MALDI TOF MS, VITEK^®^ 2 Compact, PCR, Biotyping, 1B/O:8

## Abstract

**Background:**

*Yersinia enterocolitica* is widespread within the humans, pigs and wild boars. The low isolation rate of *Y. enterocolitica* from food or environmental and clinical samples may be caused by limited sensitivity of culture methods. The main goal of present study was identification of presumptive *Y. enterocolitica* isolates using MALDI TOF MS. The identification of isolates may be difficult due to variability of bacterial strains in terms of biochemical characteristics. This work emphasizes the necessity of use of multiple methods for zoonotic *Y. enterocolitica* identification*.*

**Results:**

Identification of *Y. enterocolitica* isolates was based on MALDI TOF MS, and verified by VITEK^®^ 2 Compact and PCR. There were no discrepancies in identification of all human’ and pig’ isolates using MALDI TOF MS and VITEK^®^ 2 Compact. However three isolates from wild boars were not decisively confirmed as *Y. enterocolitica*. MALDI TOF MS has identified the wild boar’ isolates designated as 3dz, 4dz, 8dz as *Y. enterocolitica* with a high score of matching with the reference spectra of MALDI Biotyper. In turn, VITEK^®^ 2 Compact identified 3dz and 8dz as *Y. kristensenii*, and isolate 4dz as *Y. enterocolitica*. The PCR for *Y. enterocolitica* 16S rDNA for these three isolates was negative, but the 16S rDNA sequence analysis identified these isolates as *Y. kristensenii* (3dz, 4dz) and *Y. pekkanenii* (8dz). The wild boar’ isolates 3dz, 4dz and 8dz could not be classified using biotyping. The main bioserotype present within pigs and human faeces was 4/O:3. It has been shown that *Y. enterocolitica* 1B/O:8 can be isolated from human faeces using ITC/CIN culturing.

**Conclusion:**

The results of our study indicate wild boars as a reservoir of new and atypical strains of *Yersinia*, for which protein and biochemical profiles are not included in the MALDI Biotyper or VITEK^®^ 2 Compact databases. Pigs in the south-west Poland are the reservoir for pathogenic *Y. enterocolitica* strains. Four biochemical features included in VITEK^®^ 2 Compact known to be common with Wauters scheme were shown to produce incompatible results, thus VITEK^®^ 2 Compact cannot be applied in biotyping of *Y. enterocolitica.*

## Background

*Yersinia enterocolitica,* a causative agent of yersiniosis is widespread in an environment and animals [1]. Yersiniosis was the third most frequently reported zoonosis in the European Union in 2015, with the notification rate of 2.20 cases per 100,000 population which was 6.8% higher than in 2014 [2]. In 2014 in Poland 244 cases of yersiniosis were recorded (0.63/100,000) [3]. *Y. enterocolitica* in humans may cause symptoms, i.e. fever, abdominal pain, diarrhea, vomiting, and pathologies such as self-limiting enteritis, acute mesenteric lymphadenitis and septicemia [4]. Pigs are generally considered a symptomless reservoir for this rod shaped bacterium. The wild boars are a new tested reservoir for *Y. enterocolitica*. The wild boar population in Europe and Poland has increased and venison consumption by humans is currently higher. Growing tendency of out-door pig farming is also noted [5–7]. Wild boars appear to be new, underestimated reservoir for *Y. enterocolitica*. Therefore, studies on the prevalence of *Y. enterocolitica* in wild boars can be considered important from the point of view of public health. Bancerz-Kisiel et al. in 2015 [8] isolated *Y. enterocolitica* from 26.5% of 151 wild boars hunted during 2012/2013 season in central, northern and southern Poland and that was the first report on *Y. enterocolitica* wild boars prevalence in Poland. The research also undertaken by Bancerz-Kisiel et al. [9] indicated the isolation of *Y. enterocolitica* from the half of 20 wild boar carcasses.

Despite numerous reports on the occurrence of *Y. enterocolitica* among humans, pigs or wild boars, the detection and identification are still difficult due to an overgrowth by bacteria from poly-contaminated samples, unusual growth characteristics of *Y. enterocolitica* and arduous discrimination of its colonies from other bacteria exhibiting *Yersinia*-like colonies on cefsulodin-irgasan-novobiocin Agar (CIN) such as *Citrobacter freundii*, *C. braakii*, *Aeromonas hydrophila, Enterobacter cloacae*, *Providencia rettgeri*, and *Morganella morganii* [10–12]. The recommended isolation of *Y. enterocolitica* is carried out by using ISO 10273–2003 method [13]. This method is recommended for both food and pig tonsils analyses. This protocol involves enrichment in peptone sorbitol bile (PSB) broth, and streaking on CIN. Another enrichment is also recommended, irgasan-ticarcillin-potassium chlorate (ITC) broth and streaking on *Salmonella-Shigella*-sodium-deoxycholate-calcium chloride (SSDC) agar plate [14]. However Fondrevez [15] modified this protocol and proved that streaking on CIN Agar from ITC broth could increase the number of positive samples in comparison with ISO 10273–2003 method [15]. The ITC/CIN culturing both favors the growth of biotype 4 [16, 17] which is the most common biotype in Europe within humans and also animals such as slaughtered pigs [18].

To isolate presumptive strains of *Y. enterocolitica,* the ITC broth and CIN agar media are recommended [3, 4]. CIN agar was devised by Schiemann in 1979 and is still useful in *Y. enterocolitica* isolation [10–12, 19–21]. Morphology of *Yersinia* sp. colony on CIN agar is known as red bull’s eye, red centre with colourless translucent rim [11, 12].

Many methods have been designed for the identification of *Y. enterocolitica* [19]. In the present study we verified the Matrix-Assisted Laser Desorption Ionization-Time of Flight Mass Spectrometry (MALDI TOF MS) results using the VITEK^®^ 2 Compact system, and polymerase chain reaction (PCR) for identification of *Y. enterocolitica* isolated from humans and animal sources.

An alternative to time-consuming and biochemical-based techniques is MALDI TOF MS, a suitable, rapid and powerful tool for identification of the *Y. enterocolitica* [1, 22–26]. Some data confirmed its utility in the biotyping [27, 28]. Biomarkers such as ribosomal, structural, and DNA/RNA binding proteins produce the fingerprints that vary between different microorganisms and have spectrum specific to genus, species, and subspecies [29]. Despite the great advantages of MALDI TOF MS, there are also some limitations. The identification of environmental and potentially zoonotic strains is feasible only if the database of reference spectra contains fingerprints of genera, species or subspecies of required strains [30]. As yet MALDI TOF MS was applied mostly for clinical *Y. enterocolitica* strains [1, 24–27, 31]. Few authors report on application of MALDI TOF MS to identification of potentially zoonotic *Y. enterocolitica* strains [23, 28]. MALDI TOF MS was already used as a tool for identification of *Y. enterocolitica* strains isolated from slaughtered pigs and wild boars [32]. This work has not revealed the usefulness of MALDI TOF MS in zoonotic *Y. enterocolitica* strains identification but authors have confirmed all results using 16S rDNA sequencing [32].

VITEK^®^ 2 Compact is automatic biochemical-based method which includes 48 biochemical features, being commonly used for microbial identification in clinical laboratories [33, 34]. VITEK^®^ 2 Compact allows microorganisms’ identification up to 4 h. Each well measures the metabolic activity of strain among ability to acidification, alkalization and enzymatic hydrolysis of substrates and bacterial growth in the presence of inhibitors. The system detects bacterial growth and metabolic changes in the microwells by using a fluorescence-based technology. Results of mentioned biotyping and biochemical-based methods may be influenced by conditions of bacterial incubation such as the composition of media or pH [34]. There is no available data on the utility of VITEK^®^ 2 Compact in identification of zoonotic *Y. enterocolitica* isolates.

A number of DNA-based methods for identification of zoonotic *Y. enterocolitica* strains such as PCR, and DNA sequencing was introduced [35]. Three of them have used the 16S rDNA-specific PCR designed by [36] for accurate and rapid species confirmation of *Y. enterocolitica* [36–38] In other PCR techniques identification of *Y. enterocolitica* were based on detection of particular species-specific virulence genes [10, 19, 32, 37, 38]. The 16S rDNA sequence analysis is still the gold standard in microbial identification. In this paper 16S rDNA sequence analysis was used to reveal the species membership of three wild boars isolates.

Due to the biochemical characteristics and high heterogeneity *Y. enterocolitica* has been divided into the biotypes (Table 1) while the serotype is dependent on its lipopolysaccharide (LPS) structures. The biotype 1A is considered to be non-pathogenic to humans, whereas the biotype 1B, so-called American, is the most dangerous biotype within a species. The biotypes 2, 3, 4, 5 called European, are considered as weakly pathogenic. The most frequently used biotyping scheme was described in 1987 by Wauters [39]. Efficiency of biotyping may be limited by presence of unusual animals’ strains. Within the biochemical features of VITEK^®^ 2 Compact only four of them are included in biotyping scheme proposed by Wauters. Since serotypes belonging to different biotypes (1A, 1B, 2–5) may have a common O-specific LPS antigen, serotyping cannot be definite information on strain pathogenicity.

**Table 1:** Biochemical tests used for *Yersinia enterocolitica* biotyping based on Wauters [39, 41]

*Y. enterocolitica* biotypes pathogenicity against humans		Reaction of biotypes							
		Lipase^a^	Esculin	Indole	Xylose^a^	Trehalose^a^	Pyrazinamidase	β -D-Glucosidase^a^	Voges-Proskauer
Nonpathogenic	1A	+	+	+	+	+	+	+	+
Highly pathogenic	1B	+	–	+	+	+	–	–	+
Weakly pathogenic	2	–	–	(+)	+	+	–	–	+
	3	–	–	–	+	+	–	–	+ (c)
	4	–	–	–	–	+	–	–	+
	5	–	–	–	V	–	–	–	(+)

() = Delayed reaction; V = variable reaction; (c) Biotype of serotype O:3 found in Japan [39]; ^a^ feature included in VITEK^®^ 2 Compact

The main goal of present study was identification of presumptive *Y. enterocolitica* isolates using MALDI TOF MS. The consecutive aim was verification of MALDI TOF MS results of *Y. enterocolitica* using VITEK^®^ 2 Compact and PCR methods. Based on these results we investigated the incidence of *Y. enterocolitica* in humans and prevalence within pigs and wild boars during the years 2014–2015 in south-west Poland, where no research on this topic has been carried out yet.

## Methods

### Bacterial isolates

The bacterial isolates were obtained from 293 human faeces swabs routinely examined by PhD Kamila Korzekwa at Dialab Medical Laboratory (Wrocław, Poland). Bacteria were also isolated from 168 rectal and tonsillar swabs taken from healthy half-year old fattening pigs (*Sus scrofa domestica*), and from 130 swabs taken from tonsils and nasopharynx of hunted wild boars (*Sus scrofa*). Swabs from pigs and wild boars were taken by doctor of veterinary medicine Prof. Jarosław Bystroń at two separate slaughterhouses and one meat processing plants in Wielkopolskie voivodeship (south-west Poland). All isolates isolated from separate individuals were deposited at the Collection of Department of Microbiology, Institute of Genetics and Microbiology, University of Wrocław (Table 3).

The *Y. enterocolitica* reference strains used as a control in this study were obtained from Polish Collection of Microorganisms (PCM, Wrocław, Poland): PCM 1879 (4/O:3), PCM 1880 (5/O:3), PCM 1881 (4/O:3), PCM 1882 (1B), PCM 1883 (1A/O:5), PCM 1884 (2/O:8) and from Department of Applied Microbiology, Faculty of Biology, University of Warsaw, Poland: *Y. enterocolitica* 2/O:9.

### Isolation of *Y. enterocolitica* from environmental and clinical sources

Swabs from human faeces, half-year pigs’ tonsils or rectum and also wild boars tonsils and nasopharynx were incubated at 30 °C for 48 h in the ITC Broth (Biocorp, Warszawa, Poland) and next plated onto the CIN Agar (Biocorp, Warszawa, Poland) at 30 °C for 24 h [11]. The colonies obtained on the CIN Agar that represented the red bull’s eye morphology [12] were taken for further analysis.

### MALDI-TOF MS analyses

A bacterial suspension was made by mixing the couple of single colonies of actively growing cultures in 300 μL of water which were fixed by the addition of 900 μL absolute ethanol and followed by extraction procedure as previously described [40]. Bacterial strains were identified by MALDI-TOF MS and all analyses were performed with an UltrafleXtreme mass spectrometer (Bruker Daltonics, Germany) using the Biotyper 3.1 software and database containing 4613 entries. Identification by MALDI-TOF MS was twice repeated.

### Sample preparation and procedure of identification for VITEK^®^ 2 compact

A sterile microloop was used to take a few colonies of a pure culture cultivated on Luria-Bertani Broth (Biocorp, Warszawa, Poland) for 18 to 24 h. A bacterial suspension was adjusted to McFarland Turbidity Standard of 0.5–0.63 in 3 mL of a 0.45% sodium chloride solution with a VITEK^**®**^ 2 DensiChek (bioMérieux, Warszawa, Poland). The GN card was put on the cassette and placed in the instrument. The time interval between suspension preparation and card filling was less than 30 min to avoid changes in turbidity. Cards were incubated at 35.5 ± 1 °C. Each card was removed from the incubator every 15 min and automatically subjected for colorimetric readings. Results were read after 10 h – 18 h of incubation.

### Biotyping

The biotyping of presumptive *Y. enterocolitica* isolates *n* = 37 was carried out based on Wauters [39] with modifications [41]. Table 1 shows the scheme of reactions of the individual biotypes.

**β-D-glucosidase activity** was assayed using 0.1% 4-nitrophenyl-β-D-glucopyranoside (Sigma-Aldrich, St. Louis, MO, USA) as substrate in 0.0666 M monosodium phosphate pH 6.0 (Avantor Performance Materials, Gliwice, Poland). The bacteria suspended in 250 μL of physiologic saline to McFarland Turbidity Standard No. 3. were added to the equal volume of the substrate. Tubes were incubated at 30 °C for 24 h. Appearance of yellow colour (assessed visually) caused by nitrophenol release was interpreted as positive reaction.

**Pyrazinamidase test **was performed on slants containing tryptic soy broth 30 g/L (Biocorp, Warszawa, Poland), yeast extract 3 g/L (Biocorp, Warszawa, Poland), pyrazinecarboxanamide 1 g/L (Sigma-Aldrich, St. Louis, MO, USA), sodium chloride 5 g/L (Chempur, Piekary Śląskie, Poland) and agar 20 g/L (Biocorp, Warszawa, Poland) in 0.2 M Tris-maleate Buffer (pH 6.0). The cultures were grown on pyrazinamidase agar slants for 48 h at 30 °C. Then 1 mL of freshly prepared 1.0% aqueous solution of ferrous ammonium sulphate (Avantor Performance Materials, Gliwice, Poland) was flooded over the slants. Development of pink colour (assessed visually) within 15 min indicated presence of pyrazinoic acid formed by bacterial pyrazinamidase.

**Bromocresol purple broth** was used to assess carbohydrate fermentation. Bromocresol purple broth included proteose peptone 10 g/L (Biocorp, Warszawa, Poland), meat extract 1 g/L (Biocorp, Warszawa, Poland), sodium chloride 5 g/L (Chempur, Piekary Śląskie, Poland), bromocresol purple 0.02 g/L (Avantor Performance Materials, Gliwice, Poland) as well as xylose (Sigma-Aldrich, St. Louis, MO, USA) and trehalose (Sigma-Aldrich, St. Louis, MO, USA) at final concentration of 1.0%. Bacteria were cultured for 24 h at 37 °C in 4 mL of bromocresol purple broth. Appearance of yellow colour indicated ability of tested isolate to ferment trehalose or xylose.

**Indole production** was assessed using the tryptophan broth (Biocorp, Warszawa, Poland). Bacterial cultures were incubated for 24 h at 37 °C, each tube was then flooded by 0.2–0.3 mL Kovacs’ reagent (Pro-Lab Diagnostics, Round Rock, TX, USA). Development of deep red colour on broth surface indicated ability of indol production by tested strain.

**Voges-Proskauer test** was assayed to confirm the acetoin production by *Y. enterocolitica* strains. Bacterial cultures were incubated for 48 h at 37 °C in Clark’s medium containing glucose 5 g/L (Avantor Performance Materials, Gliwice, Poland), dipotassium phosphate 5 g/L (Chempur, Piekary Śląskie, Poland). After that 1 mL of 6.0% α-naphthol (Avantor Performance Materials, Gliwice, Poland) and 1 mL of 40% potassium hydroxide (Chempur, Piekary Śląskie, Poland) were added and shaken. The development of pink-to-ruby red colour in the medium indicated acetoin production by tested isolate.

**Lipase production** was checked on Agar medium with 1% Tween 80 (Avantor Performance Materials, Gliwice, Poland). Development of opacity in the medium indicated lipase production by tested isolate.

**Esculin hydrolysis** was detected on Bile Esculin LAB-Agar (Biocorp, Warszawa, Poland). The esculetin, which is the product of esculin hydrolysis, reacts with the ferric citrate to form a dark brown or black colony, indicating ability of isolate to hydrolyse esculin.

### Serotyping

After biotyping *Y. enterocolitica* isolates were serotyped using the commercial antisera polyvalent group O:1 O:2, O:3, O:5, O:8 and O:9 (Denka Seiken, Tokyo, Japan). Single colony of tested strain was suspended in physiological solution. A drop of antiserum was added to cell suspension and the agglutination process indicated a positive reaction between bacteria and antisera.

### DNA extraction

Total bacterial DNA from overnight culture in Luria-Bertani Broth (Biocorp, Warszawa, Poland) was extracted using commercial Genomic Mini kit (A&A Biotechnology, Gdynia, Poland) according to the manufacturer’s protocol.

### Identification of *Y. enterocolitica* using PCR targeting 16S rDNA gene

The PCR method, using specific primers for *Y. enterocolitica* 16S rDNA, i.e., Y1 5’-AATACCGCATAACGTCTTCG-3′ and Y2 5’-CTTCTTCTGCGAGTAACGTC-3′ was applied to identify all *n* = 37 isolates [36–38]. PCR was performed in a total volume of 25 μL including 1,5 μL of 25 mM MgCl_2_ (Thermo Fisher Scientific, Waltham, MA, USA), 2.5 μL of 10 × buffer (Thermo Fisher Scientific, Waltham, MA, USA), 0.5 μL of 10 mM dNTP (Thermo Fisher Scientific, Waltham, MA, USA), 0.1 μL of 100 μM primers (Genomed, Warszawa, Poland), 0.2 μL of Taq polymerase (Thermo Fisher Scientific, Waltham, MA, USA), and 1 μL of template DNA. The amplification was carried out using a MJ Mini Thermal Cycler (Bio-Rad, Hercules, CA, USA) as follows: initial denaturation at 94 °C for 5 min, followed by 30 cycles of denaturation (94 °C, 30 s), annealing at 55 °C for 30 s, and extension at 72 °C for 45 s. DNA from reference *Y. enterocolitica* strains was used as positive control. DNA from reference *Staphylococcus aureus* strain was used as negative control.

### 16S rDNA sequencing analysis

The fragments of 16S rDNA genes were amplified from bacterial DNA of strains 3dz, 4dz, 8dz and positive control *Y. enterocolitica* 2/O:9 using PCR with universal primers 16S 27f- (5′-AGAGTTTGATCMTGGCTCAG-3) [42] and 16S 519r (5′-GwATTACCGCGGCkGCTG-3′) [43] (Genomed, Warsaw, Poland), producing an amplicon of about 500 bp. PCRs were performed using 1 μL of template, 2.5 μL of 10 × Dream Taq PCR buffer (Thermo Fisher Scientific, Waltham, MA, USA), 0.1 μL of 100 μM primers (Genomed, Warsaw, Poland), 0.5 μL of 100 mM dNTP (Thermo Fisher Scientific, Waltham, MA, USA), 0.5 U of Dream Taq DNA polymerase (Thermo Fisher Scientific, Waltham, MA, USA), and sterile water added to a volume of 25 μL. Amplification was performed using a MJ Mini Thermal Cycler (Bio-Rad, Hercules, CA, USA). Thermocycling parameters were 94 °C for 2 min, 25 cycles of 94 °C for 30 s, 50 °C for 30 s and 72 °C for 30 s; and a final extension step at 72 °C for 5 min. The products of PCR amplification were examined by gel electrophoresis, and stained with ethidium bromide. The amplicons were sequenced using Sanger capillary sequencing method (Genomed, Warsaw, Poland). The sequences were identified at species level using BLAST algorithm and NCBI GeneBank database. The following criteria were applied on sequence analysis: (i) isolate was identified at species level when its sequence had a similarity score of ≥99% with that of a reference GeneBank sequence, (ii) isolate was identified at genus level when the similarity score was < 99% and ≥ 95%, and (iii) isolate was identified at family level when the similarity score was < 95%.

### Electrophoretic analysis of PCR products

The amplified products and GeneRuler DNA Ladder Mix (Thermo Fisher Scientific, Waltham, MA, USA) were resolved on 1.5% agarose gel (Prona Agarose Basica Le GQT) containing 0.5 μg/mL ethidium bromide and documented using GelDocXR System (Bio-Rad, Hercules, CA, USA). PCRs on DNA from *Y. enterocolitica* yielded a PCR product of 330 bp.

### Data analysis

In MALDI TOF MS bacterial identification was performed using Biotyper 3.0 software. Identification of isolates at species level with log scores ≥2.300 were considered highly probable, log scores between 2.299 and 2.000 indicated high probability of identification at the genus level and probable identification at species level. Log scores between of 1.999 and 1.700 yielded probable identification at genus level. Scores < 1.700 were interpreted as unreliable identification.

Confidence values of isolate identification in VITEK^®^ 2 Compact system were allocated to several categories, i.e., 96–99% indicated excellent, 93–95% very good and 89–92% good identification at the species level, whereas confidence value of 85–88% indicated acceptable or low identification at the genus level. Confidence values < 85% indicated unreliable identification.

The data of both methods were expressed at point system, wherein for the correct identification at the species or genus level awarded 3 and 1 point, respectively. For incorrect or low discrimination of identification was assigned 0. The results of *Yersinia* spp. identification obtained from 3 systems (MALDI TOF MS, VITEK^®^ 2 Compact and 16S rDNA PCR) were compared by using the chi square test for independent pairs with Yates’ correction (*n* = 37). The degree of concordance between MALDI TOF MS or VITEK® 2 Compact and PCR method was analyzed by using the Spearman correlation. A *P* value of < 0.05 was considered statistically significant. Statistical analysis was performed using Statistica ver. 12 (StatSoft, Poland).

## Results

### Isolation of bacteria on ITC/CIN

The culture of analysed material on ITC and CIN media allowed to obtain 650 presumptive *Y. enterocolitica* colonies from 591 individuals (Table 3). All 650 isolates growing on CIN Agar as mannitol fermenting colonies and presenting the “red bull’s eye” morphology were subjected to further MALDI TOF MS analysis.

### MALDI TOF MS identification and *Y. enterocolitica* occurrence

MALDI TOF MS identified 37 isolates as *Y. enterocolitica*, including three human isolates, 28 pig’ and six wild boar’ isolates (Tables 2, 3). All pig and human isolates and two of six wild boar’ isolates designated as 2dz, 5dz were identified using MALDI TOF MS with log scores ranging from 2.300 to 3.000 meaning highly probable species identification. The log score value of MALDI TOF MS for other wild boar’ isolates designated as 3dz, 4dz, 8dz, 10dz ranged from 2.000 to 2.299 indicated secure genus identification, and probable species identification.

**Table 2:** *Yersinia* sp. isolates *n* = 37 used in this study and deposited at the Collection of Department of Microbiology, Institute of Genetics and Microbiology, University of Wrocław

Source of isolates		List of strains
Dialab Medical Laboratory	Human faeces	3d, 42d, 58d
Slaughterhouse	Slaughtered pigs	90z, 119z, 121z, 163z, 165z, 168z, 169z, 170z, 171z, 173z, 174z, 175z, 176z, 177z, 179z, 180z, 182z, 183z, 184z, 186z, 187z, 188z, 189z, 190z, 191z, 192z, 193z, 194z
Meat processing plant	Wild boars	2dz, 3dz, 4dz, 5dz, 8dz, 10dz

**Table 3:** *Yersinia* sp. isolates isolation with ITC/CIN from wild boars, pigs and human’s faeces in south-west Poland during 2014–2015, identified using MALDI TOF MS and bioserotyping

Source of isolates	Wild boars		Slaughtered pigs		Humans
Type of swabs samples	Tonsils	Nasopharynx	Rectum	Tonsils	Faeces
Number of individuals	10	120	36	132	293
Number of presumptive “red bull’s eye” colonies on CIN Agar	10	133	58	145	304
Number of positive *Yersinia* sp. samples / individuals (%)	6 (6 of 10)	0 (0)	25 (69.4)	3 (2.3)	3 (1)
Bioserotype	1A/O:9 *n* = 1	–	4/O:3	4/O:3	4/O:3 *n* = 2
	1A/NT *n* = 2		*n* = 25	*n* = 3	1B/O:8 *n* = 1
	NT/NT *n* = 3				

*NT* – nontypeable

Despite the recommendation of using ITC/CIN media for selective isolation of *Y. enterocolitica* growth and “red bull’s eye” morphology of bacterial species other than *Y. enterocolitica* was observed. Other species identified using MALDI TOF MS included *Rahnella aquatilis*, *Providencia rettgeri, Erwinia persicina*, *Serratia liquefaciens, Ewingella americana, Raoultella ornithinolytica*. The most common non-*Y. enterocolitica* specimen was *Aeromonas salmonicida ssp. salmonicida.* Interestingly, all isolates isolated from wild boars, identified as *Y. enterocolitica* presented viscous and mucous morphology not observed in *Y. enterocolitica* isolates isolated from human stool or slaughtered pig tonsils.

Three of 293 human individuals were positive (1.0%) for *Y. enterocolitica.* An isolate named, as 3d and 42d were isolated from stool of 5-year old boy and 2-year old boy, respectively. An isolate named, as 58d (1B/O:8) was isolated from stool of 69-year old women. Their medical order was general examination of faeces. According to the MALDI TOF MS results six *Yersinia* sp. isolates were isolated from *n* = 10 wild boars tonsils. In the wild boars’ nasopharynx (*n* = 120) were not confirmed any of *Yersinia* sp. isolates. The number of pig’ rectum swabs (*n* = 36) was the source for 25 *Y. enterocolitica* isolates (69.4% of number of rectum’ swabs). The pig’ tonsils swabs (*n* = 132) were the source for 3 *Y. enterocolitica* isolates (2.3% of number of tonsils’ swabs) (Table 3).

### VITEK^®^ 2 compact identification of *Y. enterocolitica* isolates

All 37 isolates identified by MALDI TOF MS as *Y. enterocolitica* were then verified by VITEK^**®**^ 2 Compact. Results obtained from VITEK^®^ 2 Compact (all ranked excellent) agreed with MALDI TOF MS results for three human isolates, 28 pigs’ isolates and four wild boar’ isolates, i.e., 2dz, 4dz, 5dz and 10dz (*p* = 0.061).

However, two wild boar’ isolates identified by MALDI TOF MS as *Y. enterocolitica*, i.e., 3dz and 8dz were identified by VITEK® 2 Compact as *Y. kristensenii* (Table 4) with excellent and very good confidence, respectively.

**Table 4:** The differences between results obtained by independent methods: MALDI TOF MS, VITEK® 2 Compact, PCR with specific for *Y. enterocolitica* 16S rDNA gene and 16S rDNA sequencing. Wild boar’ *Yersinia* sp*.* isolates *n* = 3

Strain no.	Bioserotype	MALDI TOF Biotyper	VITEK^®^ 2 Compact bioMérieux	PCR targeting 16S rDNA gene of *Y. enterocolitica*	16S rDNA Sequencing Analysis
3dz	NT	*Yersinia enterocolitica*	*Yersinia kristensenii*	–	*Yersinia kristensenii*
4dz	NT	*Yersinia enterocolitica*	*Yersinia enterocolitica*	–	*Yersinia kristensenii*
8dz	NT	*Yersinia enterocolitica*	*Yersinia kristensenii*	–	*Yersinia pekkanenii*

*NT*: nontypeable

-: lack of amplification product

### PCR-identification of *Y. enterocolitica* isolates

PCRs targeting *Y. enterocolitica*-specific 16S rDNA produced a 330-bp product for 34 isolates identified previously as *Y. enterocolitica* using both, MALDI TOF MS (*P* = 0.31) and VITEK^®^ 2 Compact (*P* = 0.61). However, three isolates isolated form wild boars, i.e., 3dz, 4dz, 8dz, and identified using both, MALDI TOF MS and VITEK^®^ 2 Compact have not produced a 330-bp product in PCR. 16S rDNA sequencing analysis of these three strains (Table 4) revealed with a similarity score of ≥99% with a reference GeneBank sequence that 3dz and 4dz are *Y. kristensenii,* and 8dz is *Y. pekkanenii.*

### Bioserotyping of *Y. enterocolitica* isolates

Identification by VITEK^®^ 2 Compact based on 48 biochemical features gave results for four reactions in the biotyping scheme. However biotyping was also performed on 37 isolates according to Wauters scheme [39]. Results of incubation using Wauters methodology and results of VITEK^®^ 2 Compact differed in xylose fermentation for all wild boar’ isolates and one human isolate. VITEK^®^ 2 Compact did not detect lipase production however detected the β-D-glucosidase production of 8dz wild boar’ isolates. The differences in reactions were listed in Table 5.

**Table 5:** The differences in biochemical reaction results of selected *Yersinia* sp*.* isolates according to the Wauters scheme [39] and VITEK® 2 Compact

Strain no.	Reaction of biotypes based on Wauters methodology				Reaction of biotypes based on VITEK®2 Compact results			
	Lipase	Xylose	Trehalose	β-D-glucosidase	Lipase	Xylose	Trehalose	β-D-glucosidase
58d	+	+	+	–	–	–	+	–
2dz	+	+	+	+	–	–	+	+
3dz	–	+	+	–	–	–	+	–
4dz	–	+	+	–	–	–	+	–
5dz	+	+	+	+	–	–	+	+
8dz	–	+	+	–	–	–	+	+
10dz	+	+	+	+	–	–	+	+

All pig’ *Y. enterocolitica* isolates together with two of three human’ isolates were assigned to the biotype 4. All these isolates were identified as O3 serogroup. Remaining human *Y. enterocolitica* isolate was assigned to bioserotype 1B/O:8. Only 1 *Y. enterocolitica* isolate from wild boar’ tonsils (2dz) could be bioserotyped as 1A/O:9. Two wild boar’ isolates (5dz, 10dz) were assigned to 1A biotype but were nontypeable using antisera, while three other wild boar’ isolates (3dz, 4dz, 8dz) could not be bioserotyped (Table 3).

## Discussion

In recent years incidence of yersiniosis in Poland has remained at a stable level, but data about number of cases can be affected by low reportability, lack of bioserotyping as a part of routine diagnostics and underreporting of extraintestinal yersiniosis. In Poland prevalence of *Y. enterocolitica* in animals was studied in the north-east region of the country only [8, 19, 44, 45].

*Y. enterocolitica* has been isolated all over the world not only from pigs, but also from wild boars, sheep, horses, cattle, dogs, cats, ducks, amphibians and even beavers [10, 44–48]. The research of Bancerz-Kisiel [8] indicated the presence of *Y. enterocolitica* in 26, 5% of 151 wild boars, thus game animals were considered a potential reservoir of this pathogen. The role of wild boar, belonging together with pig to the same species (*Sus scrofa*), in *Y. enterocolitica* carriage is poorly understood [49]. In contrast, the presence of *Y. enterocolitica* in pigs is observed all over the world, accounting for increasing problem in food hygiene. We revealed that wild boars could be considered a source of *Y. enterocolitica.* However, due to some specific characteristics identification of *Y. enterocolitica* isolates from wild boars needs more effort than that from other sources.

There are many concerns on the usefulness of recommended methods for isolation and identification of *Y. enterocolitica* [5, 12, 50, 51]. Due to the overgrowth by surrounding microflora selective enrichment and culturing are needed. Isolation method using ITC/CIN media is recommended, but their sensitivity and discriminatory power are lower compared to other methods. This method takes at least 72 h to obtain presumptive *Y. enterocolitica* colonies on CIN agar. In turn, this method is much faster as compared to cold enrichment which usually includes 3-weeks incubation at 4 °C. Isolation of *Y. enterocolitca* using ITC/CIN has some limitations because many other species such as certain mannitol-fermenting bacteria can efficiently grow on these media. Moreover it is well known that ITC/CIN culturing favours the growth of biotype 4. In this work we report successful isolation of *Y. enterocolitca* bioserotype 1B/O:8 from ITC/CIN. The identification of isolates may be difficult due to variability of bacterial strains in terms of biochemical characteristics [5]. In this work the MALDI TOF MS was used in order to identify presumptive *Y. enterocolitica* isolates. To confirm identification results of potentially zoonotic isolates we used VITEK® 2 Compact, bioserotyping, and PCR.

MALDI TOF MS has been shown to be a common method in *Y. enterocolitica* identification [1, 26–28]. However, the use of both, MALDI TOF MS as well as VITEK® 2 Compact may have limitations since resources they rely on are dominated by the data from clinical isolates. Some authors reported the consistence of MALDI TOF MS results with Vitek 2 for *Y. enteroclitica* identification, but most of this research was also conducted on clinical isolates [25]. MALDI TOF MS and VITEK® 2 Compact can be thus considered first-line method for identification of *Y. enterocolitica* isolates in clinical microbiology but its use is still developing in identification of zoonotic isolates. Zoonotic *Y. enterocolitica* isolates used in this study are characterized by low diversity. Each method applied by us gave reliable and repeatable results, however they produced different results from each other especially for wild boar’ isolates. We thus included a PCR-based method, targeting 16S rDNA gene, and compared to the results by MALDI TOF MS and VITEK® 2 Compact. For most *Y. enterocolitica* isolates we demonstrated high agreement of identification results using 3 above methods. However, 3 isolates from wild boars identified as *Y. enterocolitica* by MALDI TOF MS*,* could not produce species-specific PCR amplicon for 16S rDNA gene*.* Based on 16S rDNA sequencing analysis these isolates were assigned to species other than *Y. enterocolitca*. These results indicate the necessity of using multiple approaches for identification of *Y. enterocolitica* from wild boars. These animals are generally considered an important reservoir of pathogenic *Yersinia* species [7] but in this work only nonpathogenic *Y. enterocolitica* isolates were isolated from wild boars, as inferred from biotyping results. The recent report of Magistrali et al. [52] has revealed that wild boars could act as a reservoir of atypical *Y. pseudotuberculosis,* other enteropathogenic species within the *Yersinia* genus, which were associated with severe symptoms and death of deer and macaques. As mentioned above, other probable cause of isolate misidentification can be overrepresentation of clinical strains in databases. Although these methods are often used in environmental microbiology and databases are successively expanding. Fredriksson-Ahoma et al. [6] has reported some data about the differences between *Y. enterocolitica* isolated from wild boars and domestic pigs. The differences were related to distribution of bioserotypes, virulence genes content, genotype and sensitivity to antimicrobials. In this paper [6] it was also suggested that more wild boar’ isolates have to be characterized to verify similarity between *Y. enterocolitca* from wild boars and pigs. Based on mentioned reports [6, 7, 52] we suppose that wild boars can harbor specific *Yersinia* population which may pose difficulties to standard identification.

In this work we have proven that application of single method is not sufficient for reliable identification of *Y. enterocolitica*. The discrepancy in species identification noted for certain wild boar isolates may indicate that *Yersinia* sp*.* and *Y. enterocolitica* identifiaction may need further verification. The need for application of multiple methods was already noted for the *E. coli* identification [34].

The Wauters’ scheme reactions were conducted individually. We did not considered results obtained from VITEK® 2 Compact to biotyping of our isolates, because its results were incompatible with Wauters’ scheme (Table 5). The biotyping of pig’ isolates proved the presence of *Y. enterocolitica* strains pathogenic for humans, which is consistent with the European reports [2].

Our data revealed that prevalence of *Y. enterocolitica* was higher in rectal swabs as compared to the tonsillar swabs (Table 3). It could be hypothesized that pigs examined here were in the early infection stage, when bacteria were only locally disseminated. The late infection stage is characterized by *Y. enterocolitica* presence within the lymphoid tissue e.g. tonsils, when bacteria spread through the organism [53]. In pigs slaughtered at the age of 135 days or more, the tonsils may be a more significant source of *Y. enterocolitica* than faeces [54, 55]. Pigs tested in this work were at the age of 180 days, which does not support the above hypothesis. To characterize the carrier state in pigs it is necessary to confirm if during the sampling pigs were infected systemically or locally. In addition the younger pigs should be examined for *Y. enterocolitica* occurrence.

## Conclusion

MALDI TOF MS can be successfully used for *Yersinia* sp. identifiaction from human’ and pig’ samples, but identification of wild boar’ isolates needs additional tests. The biochemical tests included in VITEK® 2 Compact are not suitable for biotyping of *Y. enterocolitica. Yersinia* sp. isolates from wild boar appear to be more heterogenous than isolates from previously characterized sources i.e. humans and pigs. Thus, the results of the present study strengthen the necessity of use of multiple methods for identification of zoonotic *Y. enterocolitica*. We found that pathogenic 1B and 4/O:3 isolates of *Y. enterocolitica* are present in south-west Poland within humans and pigs.

## Abbreviations

CIN: Cefsulodin-irgasan-novobiocin
ITC: Irgasan-ticarcillin-potassium chlorate
LPS: Lipopolysaccharide
MALDI TOF MS: Matrix Assisted Laser Desorption Ionization Time of Flight Mass Spectrometry
PCR: Polymerase chain reaction
SSDC: *Salmonella*-*Shigella*-sodium-deoxycholate-calcium chloride
